# Optimized three-level quantum transfers based on frequency-modulated optical excitations

**DOI:** 10.1038/s41598-020-59046-8

**Published:** 2020-02-10

**Authors:** Francesco Petiziol, Ennio Arimondo, Luigi Giannelli, Florian Mintert, Sandro Wimberger

**Affiliations:** 1 Department of Mathematical, Physical and Computer Sciences, University of Parma, Parco Area delle Scienze 7/A, 43124 Parma, Italy; 2 National Institute for Nuclear Physics (INFN), Milano Bicocca Section, Parma Group, Parco Area delle Scienze 7/A, 43124 Parma, Italy; 3 Department of Physics, University of Pisa, Largo Bruno Pontecorvo 3, 56127 Pisa, Italy; 4 INO-CNR, Via G. Moruzzi 1, 56124 Pisa, Italy; 5 Theoretische Physik, Universität des Saarlandes, 66123 Saarbrücken, Germany; 6 Department of Physics, Imperial College, SW7 2AZ London, United Kingdom

**Keywords:** Quantum physics, Quantum mechanics, Quantum optics

## Abstract

The difficulty in combining high fidelity with fast operation times and robustness against sources of noise is the central challenge of most quantum control problems, with immediate implications for the realization of quantum devices. We theoretically propose a protocol, based on the widespread stimulated Raman adiabatic passage technique, which achieves these objectives for quantum state transfers in generic three-level systems. Our protocol realizes accelerated adiabatic following through the application of additional control fields on the optical excitations. These act along frequency sidebands of the principal adiabatic pulses, dynamically counteracting undesired transitions. The scheme facilitates experimental control, not requiring new hardly-accessible resources. We show numerically that the method is efficient in a very wide set of control parameters, bringing the timescales closer to the quantum speed limit, also in the presence of environmental disturbance. These results hold for complete population transfers and for many applications, e.g., for realizing quantum gates, both for optical and microwave implementations. Furthermore, extensions to adiabatic passage problems in more-level systems are straightforward.

## Introduction

The near-term development of quantum technologies is fundamentally linked to the challenge of controlling quantum systems with high precision. Ideally, precision should be further flanked by flexibility and resilience against experimental imperfections, and by the capability of realizing many control operations on the system within its natural lifetime. Within the field of quantum control, a class of methodologies which satisfies many of these requirements – especially precision, flexibility and robustness – exploits the concept of adiabaticity^1^: if a system, initially prepared in an energy eigenstate, is slowly driven in time with the instantaneous eigenvalues never crossing throughout the evolution, then it always remains close to the corresponding instantaneous eigenvector of the time-dependent Hamiltonian. It follows that, if the evolving eigenvector connects the initial state to a certain target final state, adiabatic tracking produces the desired quantum state transfer.

Despite the many advantages offered by the adiabatic methods, they intrinsically suffer from the requirement of slow evolutions, which drastically limits the number of operations to be performed on the system before environmental noise spoils its quantum features. Recently, so-called counterdiabatic (cd) or shortcut-to-adiabaticity or transitionless methods^2–4^, have been explored in great detail both from the theoretical point of view and from the experimental community. Indeed, they have been implemented in two and three-level systems in different experimental contexts such as cold atoms^5–7^, trapped ions^8^, N-V centers^9–12^, superconducting qubits^13–16^, and micromechanical oscillators^17,18^. While most experiments rely on the system driven by optical excitations, superconducting qubits can exploit the additional flexibility offered by microwave excitations. The aim of these protocols is to produce the same population transfer as that given by a truly adiabatic evolution but in a shorter time. The price to pay for the speedup is typically an increase of the needed control resources^19,20^, especially in terms of tuneable Hamiltonian terms. This is in most cases too demanding from the experimental point of view: complete time-dependent control of all the couplings may be needed, for few-level systems, or even of interactions between distant subsystems for more complex systems. In order to overcome the experimental difficulty, it was soon realized that, via appropriate time-dependent unitary transformations, the scheme can in general be modified in such a way that the correcting Hamiltonian contains only realizable terms^21–25^. Physically, this typically means that the concept of adiabaticity is abandoned, and even if initial and final states are the desired instantaneous eigenstates of the original Hamiltonian, transitions are allowed throughout the intermediate path.

In this contribution, we introduce a cd scheme for producing state transfers in three-level systems which consists in an accelerated STImulated Raman Adiabatic Passage (STIRAP) built upon a general framework recently proposed^26^. This is based on the application of radiofrequency sidebands to each optical excitation of the three-level system and it will be denoted as frequency-modulated STIRAP (fmod-STIRAP). For a given modulation frequency, the only control parameter is the time-dependent amplitude of the sidebands. The key advantage of the method is that of joining very high fidelities with fast timescales, close to the quantum speed limit without needing *ad hoc* adaptations of the standard STIRAP experimental control setup. Given the wide-spread use of STIRAP protocols, the present technique is applicable to a broad range of experimental systems. Since it does not require the system to completely abandon the adiabatic path, the method maintains the robustness of STIRAP while dramatically enlarging the parameter regions in which STIRAP is efficient. Moreover, the shortcut can be straightforwardly adapted to the implementation of quantum gates via fractional STIRAP^27^, and it is not limited to a specific temporal regime: it can be beneficially used at all timescales from a quasi-adiabatic to a fully nonadiabatic regime. Finally, as compared to similar methods proposed theoretically^28,29^ and also realized in the lab^15^, the present sideband protocol does not produce diagonal terms in the system Hamiltonian, and hence it does not require to compensate variations of dynamical phase introduced by the control-induced ac-Stark shifts.

## Results

### Three-level protocols

#### STIRAP

**Figure 1:**
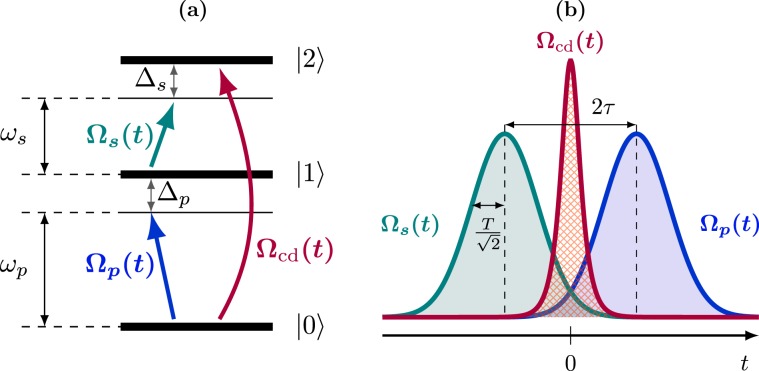
STIRAP and cd-STIRAP: ladder configuration and temporal dependence of the laser pulses. (**a**) Three-level configuration and driving scheme for the STIRAP protocol. A Stokes pulse at frequency ${\omega }_{s}$ drives the $|1\rangle$-$|2\rangle$ transition with a detuning ${\Delta }_{s}$ and Rabi coupling ${\Omega }_{s}(t)$, while a pump pulse at frequency ${\omega }_{p}$ and Rabi coupling ${\Omega }_{p}(t)$ drives the $|0\rangle$-$|1\rangle$ transition with a detuning ${\Delta }_{p}$; counterdiabatic driving requires a direct $|0\rangle$-$|2\rangle$ coupling of Rabi frequency ${\Omega }_{{\rm{cd}}}(t)$. **(b)** Sketch of the pulse shapes and of the driving sequence; the STIRAP pulses are Gaussians of standard deviation $T/\sqrt{2}$. The Stokes pulse is applied first, realizing the so-called counterintuitive sequence, followed by a pump pulse delayed by $2\tau$. While the two pulses cross at $t=0$, the counterdiabatic correction connecting states $|0\rangle$ and $|2\rangle$ reaches its maximum.

The starting point of our analysis is the three-level STIRAP protocol in ladder or $\Lambda$ configuration [Fig. 1a for the ladder one]. The STIRAP model involves three quantum states $|\mathrm{n}\rangle$ with energies ${E}_{n}$, $n=\mathrm{0,1,2}$, whose $|0\rangle$-$|1\rangle$ and $|1\rangle$-$|2\rangle$ transitions are driven by external optical/microwave fields. The essence of this process is that of producing an optical/microwave population transfer from the initial state $|0\rangle$ to the final state $|2\rangle$, which cannot be coupled directly by an electric-dipole transition, without populating the intermediate state $|1\rangle$. The STIRAP process is realized by driving the system in time in such a way that it follows as closely as possible a certain instantaneous eigenstate of the driven Hamiltonian, called the dark state, which adiabatically connects the $|0\rangle$ and $|2\rangle$ states with no overlap over state $|1\rangle$ throughout the evolution. The features which make STIRAP very interesting are especially its insensitivity against losses by spontaneous emission from the $|1\rangle$ state, and its robustness against experimental imperfections in the pulses, such as shape, timing or intensity^30^. The driving scheme involves a first “Stokes” pulse on the $|1\rangle$-$|2\rangle$ transition at frequency ${\omega }_{s}$ with time-dependent Rabi coupling ${\Omega }_{s}(t)$, and a second “pump” pulse on the $|0\rangle$-$|1\rangle$ transition at frequency ${\omega }_{p}$ and envelope ${\Omega }_{p}(t)$, respectively, slightly delayed in time, see Fig. 1. The atomic Hamiltonian in units of $\hslash$, in a frame rotating at both the driving frequencies and in the rotating-wave approximation, reads

1 $${H}_{{\rm{s}}{\rm{t}}}(t)=\frac{1}{2}(\begin{array}{ccc}0 & {\Omega }_{p}(t) & 0\\ {\Omega }_{p}(t) & 2{\Delta }_{p} & {\Omega }_{s}(t)\\ 0 & {\Omega }_{s}(t) & 2\delta \end{array}).$$

The quantities ${\Delta }_{p}={\omega }_{p}-({E}_{1}-{E}_{0})$ and ${\Delta }_{s}={\omega }_{s}-({E}_{2}-{E}_{1})$ denote the detunings of the pump and Stokes pulses from the corresponding transitions, whilst $\delta ={\Delta }_{p}+{\Delta }_{s}$ denotes the two-photon detuning for the $|0\rangle$-$|2\rangle$ transition. STIRAP works under the resonance condition $\delta =0$, and it can be realized on the basis of different temporal dependencies for the Stokes and pump pulses^28^. The present analysis is restricted to Gaussian pulses, typically used for STIRAP^30^, of the form

2 $${\Omega }_{p}(t)={\Omega }_{0}{e}^{-{(\frac{t-\tau }{T})}^{2}},\,{\Omega }_{s}(t)=\kappa \,{\Omega }_{0}{e}^{-{(\frac{t+\tau }{T})}^{2}},$$

here parametrized such that they cross at time $t=0$, with $\tau  > 0$. Therefore, the pulses have standard deviation $T/\sqrt{2}$, are delayed in time by $2\tau$, and their peak values are proportional to each other via the dimensionless parameter $\kappa$ [Fig. 1b]. In the following, we will consider the case of equal peak value of the pulses, $\kappa =1$. For maintaining the treatment general with respect to the choice of experimental context, we will work with the dimensionless quantities^31^ ${t}_{T}=t/T$, ${\Omega }_{0T}={\Omega }_{0}T$, ${\tau }_{T}=\tau /T$. The STIRAP Hamiltonian (1) has one instantaneous eigenstate, the dark state $|D(t)\rangle$^32,33^, which evolves only in the $|0\rangle$-$|2\rangle$ subspace, with no overlap over the intermediate state $|1\rangle$. That state is defined as

3 $$|D(t)\rangle =\,\cos \,\theta (t)|0\rangle -\,\sin \,\theta (t)|2\rangle ,$$

where the mixing angle $\theta (t)$ is given by $tan\theta (t)={\Omega }_{p}(t)/{\Omega }_{s}(t)$. If the Rabi couplings of the pump and Stokes pulses in the STIRAP Hamiltonian (1) are varied sufficiently slowly^30^, the system initially prepared in the ground state $|0\rangle$ will adiabatically follow the instantaneous dark state, ending up in the target state $|2\rangle$. In terms of the mixing angle $\theta (t)$, this corresponds to a variation from $\theta ({t}_{i})\simeq 0$ to $\theta ({t}_{f})\simeq \pi \mathrm{/2}$. A satisfactory adiabatic following requires rather long timescales.

*cd-STIRAP*. Among possible shortcuts to adiabaticity, much attention has been given to the idea of cd driving^19^, also known as transitionless quantum driving^20^. This protocol consists in applying additional control fields which compensate for nonadiabatic transitions exactly by instantaneously decoupling the adiabatic states. As a result, the latter are followed with unit fidelity at all times. The price to pay for this precision is the necessity to realize new time-dependent couplings in the Hamiltonian: indeed, counterdiabatic STIRAP (cd-STIRAP) requires a direct control on the $|0\rangle$-$|2\rangle$ transition^19,34^. Specifically, for resonant STIRAP (i.e., ${\Delta }_{p}=\delta \mathrm{=0}$), the correcting Hamiltonian ${H}_{{\rm{cd}}}(t)=i{\Omega }_{{\rm{cd}}}(t\mathrm{)/2}[|0\rangle \langle 2|-|2\rangle \langle 0|]$, with Rabi frequency

4 $${\Omega }_{{\rm{cd}}}(t)=2{\partial }_{t}\theta (t)=2\frac{{\Omega }_{s}(t){\partial }_{t}{\Omega }_{p}(t)-{\Omega }_{p}(t){\partial }_{t}{\Omega }_{s}(t)}{{\Omega }_{s}^{2}(t)+{\Omega }_{p}^{2}(t)},$$

is purely imaginary and completely off-diagonal in the atomic eigenbasis^19,28,29,34^. For the Gaussian pulses of Eq. (2), the Rabi frequency of Eq. (4) explicitly reads

5 $${\Omega }_{{\rm{c}}{\rm{d}}}(t)=4\frac{\tau }{{T}^{2}}\frac{1}{\cosh \,(4\frac{t\tau }{{T}^{2}})}=\frac{{\Omega }_{{\rm{c}}{\rm{d}}}^{{\rm{p}}{\rm{e}}{\rm{a}}{\rm{k}}}}{\cosh ({\Omega }_{{\rm{c}}{\rm{d}}}^{{\rm{p}}{\rm{e}}{\rm{a}}{\rm{k}}}t)},$$

with peak value ${\Omega }_{{\rm{cd}}}^{{\rm{peak}}}\mathrm{=4}\tau /{T}^{2}$ and the temporal dependence in Fig. 1b.

#### fmod-STIRAP

Now, we present the new shortcut method which aims at achieving counterdiabatic driving without requiring control of the $|0\rangle$-$|2\rangle$ direct coupling. Indeed, the fmod-STIRAP protocol attains a speed up of the dark state adiabatic state transfer by adding dynamical corrections to the pump and Stokes pulses. The fmod-STIRAP control Hamiltonian, derived in the Methods section, is

6 $${H}_{{\rm{f}}{\rm{m}}{\rm{o}}{\rm{d}}}(t)={\Omega }_{d}(t)(\begin{array}{ccc}0 & {\rm{c}}{\rm{o}}{\rm{s}}\,({\omega }_{d}t+{\phi }_{G}) & 0\\ {\rm{c}}{\rm{o}}{\rm{s}}({\omega }_{d}t+{\phi }_{G}) & 0 & \sin \,({\omega }_{d}t+{\phi }_{G}+{\phi }_{R})\\ 0 & \sin \,({\omega }_{d}t+{\phi }_{G}+{\phi }_{R}) & 0\end{array}),$$

where we introduce also the global phase ${\phi }_{G}$ and the relative phase ${\phi }_{R}$ in order to test the robustness against variations from their zero values. The Rabi frequency is

7 $${\Omega }_{d}(t)=\sqrt{{\omega }_{d}{\Omega }_{{\rm{cd}}}(t)}=\sqrt{\frac{{\omega }_{d}{\Omega }_{{\rm{cd}}}^{{\rm{peak}}}}{\cosh ({\Omega }_{{\rm{cd}}}^{{\rm{peak}}}t)}},$$

with the last equality holding for the Gaussian pulses of Eq. (2), and the peak value being ${\Omega }_{d}^{{\rm{peak}}}=\sqrt{{\omega }_{d}{\Omega }_{{\rm{cd}}}^{{\rm{peak}}}}$. This Hamiltonian can be realized by properly producing and combining the sidebands at frequencies ${\omega }_{d}$ and $-{\omega }_{d}$ having amplitudes ${\Omega }_{d}(t)$, as described in the Methods section. The Rabi coupling ${\Omega }_{d}(t)$ is chosen in such a way that the fmod Hamiltonian, in the limit of high frequency ${\omega }_{d}$, reproduces the cd Hamiltonian ${H}_{{\rm{cd}}}$ effectively. Therefore, the fmod-STIRAP inherits many of the interesting features of cd-STIRAP, such as the fact that the correction vanishes in the adiabatic limit^35^ and also at the beginning and at the end of the protocol. It produces a quickly-oscillating micromotion with global dynamics following the adiabatic dynamics. This protocol represents an extension of a recently proposed one with a single sideband^29^, already implemented experimentally^15^. Here, both the ${\omega }_{d}$ and $-{\omega }_{d}$ sidebands produce the required $|0\rangle$-$|2\rangle$ coupling. At large values of ${\omega }_{d}$, within an adiabatic elimination of the intermediate state $|1\rangle$, their action is described by a two-photon transition between initial and final states as studied in an earlier work^28^. The protocol has the crucial advantage that, due to its intrinsic symmetry, it does not need additional corrections to the Hamiltonian in order to compensate for the diagonal terms created by the ac-Stark shifts. Indeed, the phase relation between the two sidebands produces shifts which cancel one another to the leading order [see Methods, Eq. (12) and subsequent discussion].

### Numerical analysis

#### Population transfer

**Figure 2:**
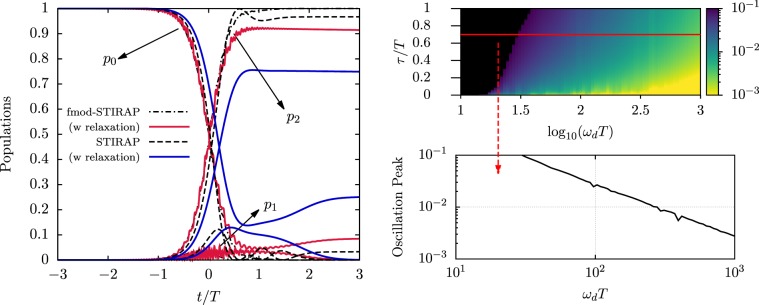
Evolution of the occupation probability and micromotion oscillations. (**a)** Left panel: Evolution of the occupations ${p}_{n}$, $(n=\mathrm{0,1,2)}$ for fmod-STIRAP, as compared to standard STIRAP, for parameters $\tau =0.7T$, ${\omega }_{d}\,T=60$, ${\Omega }_{0}T=12$. The  and  lines represent the results for the two protocols, respectively, in the absence of dissipative effects; the  and  curves include relaxation rates ${\varGamma }_{01}T=7$ and ${\varGamma }_{12}T=3\cdot {10}^{-3}$ of a Rydberg state excitation [see Methods]. **(b)** Right panel: On the top 2D color map, the peak value of the oscillations in the time-dependent infidelity is depicted as a function of ${\tau }_{T}$ and ${\omega }_{d}T$, in the absence of damping, for ${\Omega }_{0T}=20$. The lower panel shows a profile of the above 2D color map for ${\tau }_{T}=0.7$. Notice that at low values of ${\omega }_{d}T$ the protocol is not efficient (black areas indicating fidelity lower than 0.9).

Here we discuss the performance of the fmod protocol for the desired population transfer between states $|0\rangle$ and $|2\rangle$. An example of the temporal evolution of the occupation probabilities of the atomic states, with the system initialized in the $|0\rangle$ state, is in Fig. 2a, both in the ideal case (broken lines) and in the presence of relaxation (solid lines) [see Methods for the treatment of relaxation]. The protocol produces a net improvement with respect to STIRAP, which does not attain a large fidelity instead for the set of parameters chosen. The micromotion at frequency ${\omega }_{d}$ and $2{\omega }_{d}$ shows oscillations around the target values whilst disappearing at the protocol end. The maximal amplitude of such oscillations occurs for the population ${p}_{1}$, where their envelope follows the temporal dependence of the ${\Omega }_{{\rm{cd}}}(t)$ Rabi frequency. The oscillation peak determined by the $\tau$ and ${\omega }_{d}$ values decreases by increasing the latter parameter as in Fig. 2b.

**Figure 3:**
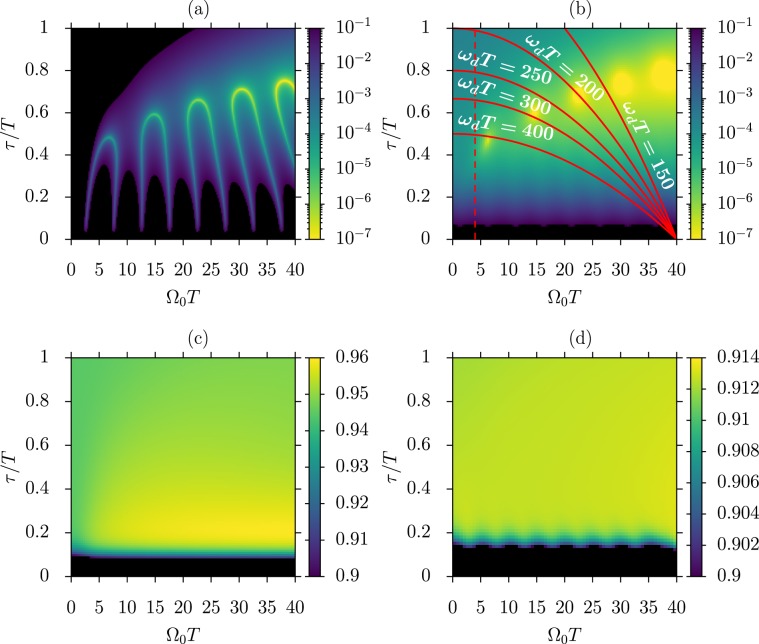
Population transfer produced by STIRAP and fmod-STIRAP for different characteristic parameters. **(a)** Final infidelity $1-{\mathbb{F}}$ for STIRAP without relaxation. **(b)** Final infidelity $1-{\mathbb{F}}$ for fmod-STIRAP of Eq. (6) with ${\omega }_{d}T=60$, ${\phi }_{G}={\phi }_{R}=0$ and without relaxation. The  lines set the boundary below which the fmod-STIRAP can be applied while satisfying the constraint on the maximal laser intensity discussed in the text, for ${\Omega }_{{\rm{in}}}T=40$ and different driving frequencies. **(c,d)** Final fmod fidelity in the presence of relaxation for the decay rates of a Rydberg optical excitation ${\Gamma }_{01}T=7$, ${\Gamma }_{12}T=0.003$ with ${\omega }_{d}T=120$ (**c**) and of a superconducting circuit ${\Gamma }_{01}T=0.017$ and ${\Gamma }_{12}T=0.023$ with ${\omega }_{d}T=35$ (**d**) [see Methods]. The stronger decay from the intermediate level of the Rydberg system, as compared to the circuit QED one, calls for a larger fmod driving frequency for attaining a given final fidelity. In all panels the black areas indicate regions where the fidelity does not reach 0.9. All data were produced by considering a time interval $[-8T,\,4T]$.

A more thorough comparison between the two protocols is shown in Fig. 3. The numerical results reported in that Figure represent the final infidelity as a function of the ${\Omega }_{0T}$ and ${\tau }_{T}$ dimensionless parameters, in the absence (3a,b) and in the presence (3c,d) of relaxation. The infidelity is defined as $1-{\mathbb{F}}(t)$, with ${\mathbb{F}}(t)={|\langle \psi (t)|2\rangle |}^{2}$ the fidelity between the system final state and the target state. While STIRAP gives optimal results in a rather small region of the parameter space (Fig. 3a), the fmod protocol produces very high fidelities almost everywhere, getting above 0.9999999 (Fig. 3b). Interestingly, the two methods give their best (though different) results for very similar parameters, such as $({\Omega }_{0T},{\tau }_{T})\simeq$ (7, 0.5), (15, 0.6), for which the adiabatic condition^30^ on the STIRAP pulses is satisfied quite well. The global behavior of the protocols with respect to the parameter variations is retained in the presence of relaxation, with the overall final fidelity settling to lower values, still above $0.9$ at the time $t/T=4$ both for the optical (Fig. 3c) and the microwave (Fig. 3d) implementation. Additional simulations (not shown here) were performed for probing the response of the protocol to situations in which decoherence acts faster, which may be of interest for applications in solid-state systems such as color centers. This has been done by introducing artificial decoherence channels (see Methods), with rates ranging within some orders of magnitudes larger than the ${\Gamma }_{12}$ rate of the Rydberg excitation. We find that dephasing rates up to $\sim \,100{\Gamma }_{12}$ in one channel still lead to fidelities above $0.9$ for the parameters used in Fig. 3c within most of the parameter space.

#### Protocol speed

**Figure 4:**
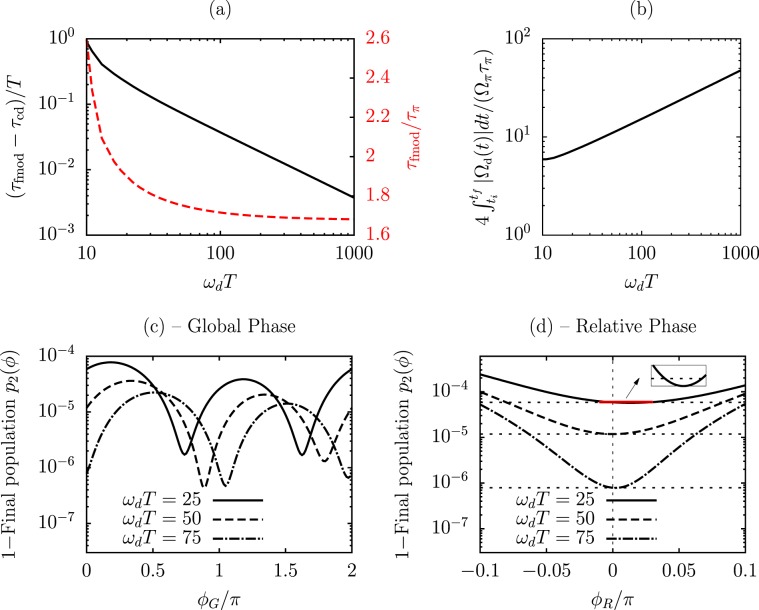
Protocol quantum speed and influence of global and relative phases between the fmod control fields. (**a)** Difference between the transfer times ${\tau }_{{\rm{fmod}}}$ for fmod-STIRAP and ${\tau }_{{\rm{cd}}}$ for cd-STIRAP in units of $\mathrm{1/}T$ ( referred to the vertical axis on the left) and ratio ${\tau }_{{\rm{fmod}}}/{\tau }_{\pi }$ as a function of the driving frequency ${\omega }_{d}T$ ( referred to the vertical axis on the right). The parameters used are ${\Omega }_{0T}=10$, ${\tau }_{T}=0.7$; **(b)** Total pulse area of fmod-STIRAP, considering the pump/Stokes sidebands, divided by the area of a pulse with constant Rabi frequency realizing the same transfer by direct $|0\rangle$-$|2\rangle$ coupling, as a function of the driving frequency. **(c)** Infidelity for the target state $|2\rangle$ at the end of the fmod-STIRAP protocol as a function of the global phase ${\varphi }_{G}$, for three different driving frequencies. **(d)** The same quantity of (c) as a function of the relative phase ${\varphi }_{R}$ between the control fields, for ${\phi }_{G}=0$. The inset shows that very small phase variations can accidentally lead to a smaller infidelity.

An important target for the applications of protocols such as cd-STIRAP and fmod-STIRAP described in this work is the possibility of speeding up, using limited resources, the quantum transfer process between initial and final states. For these protocols, we compare the transfer time from being in the state $|0\rangle$ with probability ${p}_{0}^{(i)}$ to being in the target state $|2\rangle$ with probabilty ${p}_{2}^{(f)}$, where we will take ${p}_{0}^{(i)}={p}_{2}^{(f)}=0.99$. We restrict our attention to the case of resonant laser driving where the required resources are at their minimum. Our reference point is the quantum speed limit (QSL) determined by different bounds^36^. For a start, we consider the limiting case of a $\pi$-pulse of constant Rabi frequency ${\Omega }_{\pi }$ linking directly the initial and final states. In that case, the quantum speed limit time is given by ${\tau }_{\pi }=\mathrm{2.74(1)/}{\Omega }_{\pi }$ for our initial and final occupation choice [see Methods]. Concerning standard STIRAP instead, our touchstone is the estimate obtained for Gaussian pulses in previous works^7,10,28^. Numerical analyses indicate a transfer time ${\tau }_{{\rm{st}}}=\mathrm{7.05(2)/}{\Omega }_{0}$ for pulses having optimized parameters $T=\mathrm{3.00(1)}$ and $\tau =\mathrm{0.175(5)}T$. For cd-STIRAP, where the system follows exactly the dark state of (3), the transfer time is computed analytically in the Methods section for the case of Gaussian STIRAP pulses, and it reads ${\tau }_{{\rm{cd}}}=\mathrm{4.59(5)/}{\Omega }_{{\rm{cd}}}^{{\rm{peak}}}$. The fmod-STIRAP protocol targets to reach the transfer time of cd-STIRAP. This objective is achieved by increasing the driving frequency ${\omega }_{d}T$ as presented in Fig. 4a. The fmod-STIRAP transfer time represented in this figure () is computed by performing a sigmoid fit to the temporal evolution of the populations, in order to remove the oscillatory pattern, as detailed in Methods.

#### Resources and constraints

When an increase of the driving frequency ${\omega }_{d}$ brings the fmod-STIRAP transfer time closer to the cd-STIRAP one, it also implies an increase of the Rabi frequency ${\Omega }_{d}(t)$ [(7)] which is proportional to $\sqrt{{\omega }_{d}}$. The tradeoff between time and resources can be characterized by comparing the total pulse area used by fmod-STIRAP with that of a $\pi$-pulse, see Fig. 4b and Methods, for the initial and final 0.99 occupations of interest. This treatment is also connected to definitions of intrinsic speed limits based on geometric approaches^37,38^, since the norm of the Hamiltonian is proportional to the Rabi frequency of the corresponding pulses for the present protocols. In realistic experimental implementations, upper bounds may be imposed on the strength of the correcting Hamiltonian, or more precisely on the Rabi frequency ${\Omega }_{d}^{{\rm{peak}}}$ of Eq. (7). Those bounds, with ${\Omega }_{{\rm{cd}}}$ fixed by the parameters of STIRAP, imply a limitation to the driving frequency ${\omega }_{d}$ which can be applied. Great care should be payed to the reduction of ${\omega }_{d}$ because a slow modulation does not compensate for undesired transitions and the method’s enforced adiabaticity breaks down. In alternative, the parameters of STIRAP could be tuned to a different operational point in the $({\tau }_{T},{\Omega }_{0T})$ 2D space. The important role of the parameter ${\omega }_{d}T$ appears clearly within an optical implementation of the fmod protocol where the laser intensity is distributed among the carrier with Rabi frequency ${\Omega }_{0}$ and the sidebands with maximal Rabi frequency ${\varOmega }_{d}^{{\rm{peak}}}$ [see Methods]. A maximal input intensity ${\Omega }_{{\rm{in}}}^{2}$ sets the constraint ${\Omega }_{0}^{2}+\mathrm{2(}{\Omega }_{d}^{{\rm{peak}}}{)}^{2}\le {\Omega }_{{\rm{in}}}^{2}$, which using Eqs. (7) and (5) translates into $\tau /T\le [{\Omega }_{{\rm{in}}}^{2}-{\Omega }_{0}^{2}]T\mathrm{/8}{\omega }_{d}$. This corresponds to a reduction of the $({\Omega }_{0T},{\tau }_{T})$ parameter space where the fmod method can be applied for a given driving frequency ${\omega }_{d}$, as denoted by the boundaries plotted in Fig. 3b (), obtained for ${\Omega }_{{\rm{in}}}T=40$. An additional lower bound is imposed by assuming an upper limit ${\alpha }_{m}\mathrm{=0.9}$ on the carrier-sideband conversion, corresponding to ${\Omega }_{0}\le \mathrm{10 \% }{\Omega }_{{\rm{in}}}$ – vertical dashed line () in Fig. 3b. Even if an increase of ${\omega }_{d}$ limits the accessible parameter space, an input constraint ${\Omega }_{{\rm{in}}}T=40$ still allows to operate at points where best results are attained for different values of ${\omega }_{dT}$. On the other hand, in the superconducting-circuit experiment^15^, a single sideband was produced by a separated microwave source with ${\Omega }_{d}^{{\rm{peak}}}T=8.5$ complementing the ${\Omega }_{0T}=4.5$ of STIRAP. In this case, the fmod implementation requires, for the second sideband generation, a similar and separate microwave source, which then allows STIRAP and fmod-STIRAP to work at the best operational points of Fig. 3a,b.

#### Robustness

The infidelity minima (fidelity maxima) in Fig. 3b give evidence that the fmod-STIRAP protocol is robust against variations in the control parameters. Indeed, a fidelity around 0.9999 is reached within a half the parameter space of that figure. In order to verify that the fmod procedure does not result in difficulties in explicit implementations, we also analyze the sensitivity with respect to the phases of the control fields, which may be criticized as a weakness of other shortcuts to adiabaticity^30^. In Fig. 4c,d we report numerical results which describe the dependence of the final infidelity on both the global phase ${\phi }_{G}$ and the relative phase ${\phi }_{R}$ of Eq. (6). The results show that the method exhibits small sensitivity to phase errors. First of all, Fig. 4c indicates that, even in the presence of very large global phase shifts, fidelities above 0.9999 are always attained for different values of the driving frequency ${\omega }_{d}$. One can see that minima in the infidelity may be shifted with respect to the zero value of the global phase, lying at different points for different frequencies ${\omega }_{d}T$. Raising the driving frequency brings the zero value closer and closer to being a minimum, while the fmod Hamiltonian approximates better and better the cd Hamiltonian. For intermediate-range driving frequencies, it may be convenient to exploit this analysis in order to optimize the value of ${\phi }_{G}$. Concerning the relative phase ${\phi }_{R}$, one can see from Fig. 4d that a shift within $0.1\pi$ never decreases the fidelity below 0.999. In addition, very small phase imperfections can even produce larger fidelities, as visibile from the inset. The latter effect was analyzed in a different context^39^, and it is related to the construction of the fmod Hamiltonian: while this is engineered so to cancel nonadiabatic transitions to the leading order in $\mathrm{1/}{\omega }_{d}$, for very small phase imperfections higher orders might become non-negligible, producing in some cases an accidental error compensation. These analyses confirm the stability of our protocol.

### Quantum gates

Modified STIRAP protocols have been studied for the realization of superposition states and quantum gates^29,40–43^. A fractional STIRAP^27^ (f-STIRAP) prepares the system in an arbitrary superposition state

8 $$\psi ({t}_{i})=|0\rangle \,\to \,\psi ({t}_{f})=\,\cos \,(\eta )\,|0\rangle -{e}^{-i\chi }\,\sin \,(\eta )|2\rangle .$$

by means of the application of the following laser pulses^41^

9 $${\Omega }_{p}(t)={\Omega }_{0}\,\sin \,(\eta ){e}^{-i\chi }{e}^{-{(\frac{t-\tau }{T})}^{2}},\,{\Omega }_{s}(t)={\Omega }_{0}{e}^{-{(\frac{t+\tau }{T})}^{2}}+\,\cos (\eta )\,{\Omega }_{0}{e}^{-{(\frac{t-\tau }{T})}^{2}}.$$

The corresponding counterdiabatic correction is similar to the one of Eq. (4) depicted in Fig. 1b. The fmod shortcut Hamiltonian has the same form as that given in Eqs. (6) and (7), with the Rabi frequency ${\Omega }_{d}(t)$ recalculated starting from the above pulses. For the case of $\eta =\pi \mathrm{/4}$, $\chi =0$ and other parameters of Fig. 2a, f-STIRAP and fmod–f-STIRAP lead to infidelities equal to $4\times {10}^{-3}$ and $2\times {10}^{-7}$, respectively. A full quantum gate can be implemented by combining two non-resonant [i.e., ${\Delta }_{p}\ne 0$, $\delta =0$ in Eq. (1)] f-STIRAP processes in sequence^41^ separated in time by ${\tau }_{{\rm{sep}}}$. A reversed f-STIRAP is applied first, with pump and Stokes pulses swapped with respect to Eq. (9) and with inverted pulse separation $\tau$, followed by a standard f-STIRAP as per Eq. (9). This procedure realizes the unitary rotation

10 $$U(2\eta ,\chi )=(\begin{array}{cc}cos2\eta  & {e}^{i\chi }\,\sin \,2\eta \\ -{e}^{-i\chi }\,\sin \,2\eta  & \cos \,2\eta \end{array}).$$

**Figure 5:**
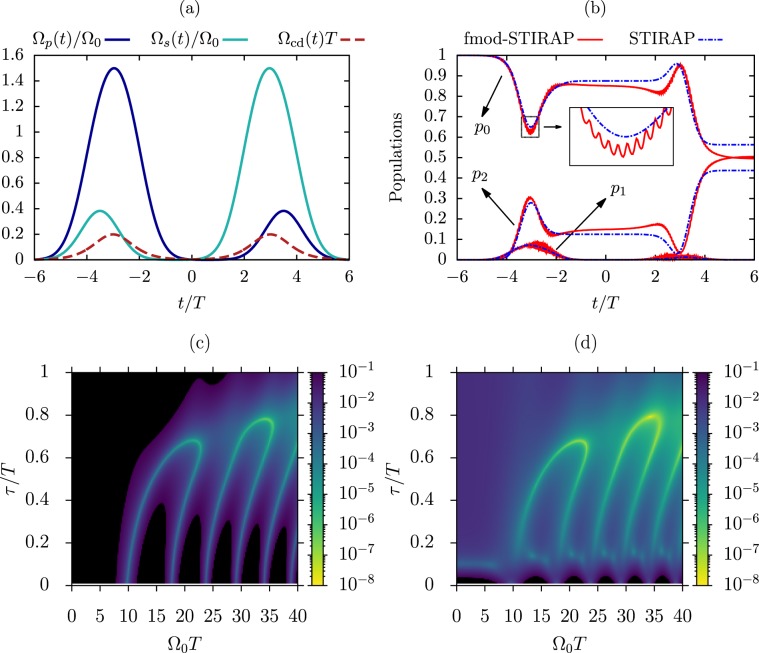
Accelerated Hadamard gate. (**a)** Temporal profile of the combined f-STIRAP pulses for the realization of a quantum gate (in dimensionless units), together with the corresponding cd Rabi frequency ${\Omega }_{{\rm{cd}}}(t)$; the parameters used, in relation to Eq. (10), are $\eta =\pi \mathrm{/8}$, and $\chi =0$. Further parameters are ${\Omega }_{0}T\mathrm{=15}$, $\tau =T\mathrm{/2}$, ${\tau }_{{\rm{sep}}}=6T$, ${\Delta }_{p}=35T$, ${\omega }_{d}T=100$; **(b)** evolution of the populations for a Hadamard quantum gate realized via the pulses shown in (a), with () and without () the fmod correction. The inset highlights the oscillating character of the dynamics of ${p}_{0}$ under fmod driving. **(c)** Final infidelity for the quantum gate as a function of the characteristic parameters of the f-STIRAP processes, in the absence of fmod correction, for the same parameters of **(b)**. **(d)** Final infidelity for the fmod–f-STIRAP protocol, for the parameters of **(b)**. Black regions in **(a)** and **(b)** indicate points where the protocols fail to surpass 0.9 fidelity.

Two f-STIRAPs are needed since a single one introduces a deleterious phase factor which is then compensated by the second f-STIRAP. The combined pulses, together with the corresponding $|0\rangle$-$|2\rangle$ counterdiabatic correction, are depicted in Fig. 5a, for the parameters in the caption. The condition ${\Delta }_{p}\ne 0$ implies that the counterdiabatic Hamiltonian ${H}_{{\rm{cd}}}(t)$ contains also $|0\rangle$-$|1\rangle$ and $|1\rangle$-$|2\rangle$ imaginary matrix elements^28,29,34^, which should be realized independently. By computing the ${\Omega }_{{\rm{cd}}}(t)$ correction separately for the two f-STIRAP protocols, the fmod-STIRAP population dynamics is represented in Fig. 5b for initial state $|0\rangle$ and parameters $2\eta =\pi \mathrm{/4,}\,\chi \mathrm{=0}$, which correspond to the Hadamard gate^44^. The other parameters are ${\Omega }_{0}T=15$, $\tau =T\mathrm{/2}$, ${\tau }_{{\rm{sep}}}=6T$, ${\Delta }_{p}=35T$, ${\omega }_{d}T=100$. Once again, the fmod shortcut induces larger final fidelities as compared to standard STIRAP, proving that the method can be used for efficiently speed up also the realization of STIRAP-based quantum gates. For instance, the final fidelity in Fig. 5b is 0.99998 for fmod-STIRAP, as compared to 0.998 for STIRAP. As for the case of the complete population transfer (Fig. 3a,b), we report in Fig. 5c,d a comparison between the gate infidelity produced by standard STIRAP (5c) and by the corresponding accelerated protocol (5d) as a function of the pump/Stokes pulse strength and pulse separation of the two f-STIRAP processes involved. The other parameters are the same of Fig. 5a,b. The difference in performance between the two methods is in line with what is observed for the full population transfer: while STIRAP fails to reach 0.9 fidelity (black areas) in a large sector of the parameter space, the shortcut reaches fidelities above 0.99 for basically all the points considered.

These results highlight the potential of the protocol for applications of interest in quantum computation and quantum simulation, where the possibility of increasing the number of gate operations of even a small fraction, or the precision of single-qubit gates, can represent a substantial gain.

## Discussion

The fmod-STIRAP protocol produces high fidelities systematically, opening a new route for the applicability of the STIRAP methodology (and modified STIRAPs) beyond the strictly adiabatic parameter regime. Crucially, the method can be easily realized experimentally, since it does not demand additional excitation resources, and it relies on the production of symmetric low-frequency sidebands. Indeed, the sideband generation requires easily accessible radiofrequency sources only, and it may take place at the expense of the laser intensity of the underlying STIRAP process. The potential production of undesired symmetric sidebands at larger frequencies is not a limitation: in the case in which their Rabi frequency can be controlled independently from that of the fundamental harmonic, they are a precious resource as discussed in the following. If they are not controllable in an independent manner, one can still adapt the construction of the effective Hamiltonian described in Methods in order to harness their effect: this would lead in general to a modified choice of the Rabi frequency ${\Omega }_{d}(t)$. Fmod-STIRAP can be used with excellent results to reduce and optimize the resources dedicated to the excitation itself. This operating regime of our protocol could be exploited to extend the applicability of STIRAP to solid-state systems or to extreme ultraviolet excitations, where the shorter natural lifetimes call for an increase of the excitation power in order to reach the pulse-area threshold for an efficient transfer. Furthermore, the transfer time attained by the protocol, close to the quantum speed limit, is independent of the laser peak values of the STIRAP driving pulses. It is determined only by their temporal parameters, together with the driving frequency, and this property makes the fmod scheme very attractive. Even at very low Rabi frequencies of the pump and Stokes control pulses, the evolution follows closely the one produced by exact counterdiabatic driving in both amplitude and time.

As benchmark figure of merit, we concentrated especially on the final fidelity, timing and stability with respect to variations in the choice of parameters. In this respect, the use of (two) sidebands at the same frequency is interesting *inter alia* for its simple implementability, producing competitive results with a modest increase of the STIRAP complexity. Nonetheless, the fmod strategy can be adapted and extended *ad hoc* in order to focus and optimize different aspects of the controlled dynamics. This can be done by controlling symmetric sidebands at higher frequency harmonics. The increase of control parameters can then be exploited for fine-tuned shaping of the effective Hamiltonian. Possible objectives of this procedure may be the minimization of the oscillations around the target adiabatic path^45^, or the robustness against selected types of external noise^46,47^. In this direction, the method may be combined with successful techniques from optimal control for carrying-out the optimization task, such as stochastic gradient (learning) algorithms^47^. All the mentioned features of fmod-STIRAP make it particularly fascinating from the experimental point of view, showing its potential for generalizations to speed up adiabatic passages in more-level configurations, and hence for successful applications in many branches of quantum science.

## Methods

### fmod-STIRAP hamiltonian

The shortcut Hamiltonian is constructed such that it reproduces ${H}_{{\rm{cd}}}(t)$ effectively, and it is engineered using a Floquet-Magnus average Hamiltonian scheme^48^, whose use for the derivation of approximate counterdiabatic fields has been recently proposed^26,49^. We consider a general form of the correcting Hamiltonian in which the $|0\rangle$-$|1\rangle$ and $|1\rangle$-$|2\rangle$ transitions are driven by fields oscillating at harmonics of a frequency ${\omega }_{d}=2\pi /{T}_{d}$ which is large with respect to the characteristic frequency scales of the system. The correcting Hamiltonian has the form

11 $${H}_{{\rm{f}}{\rm{m}}{\rm{o}}{\rm{d}}}(t)=\frac{1}{2}(\begin{array}{ccc}0 & f(t) & 0\\ {f}^{\ast }(t) & 0 & g(t)\\ 0 & {g}^{\ast }(t) & 0\end{array})$$

with control functions chosen as $f(t)={\sum }_{k\ne 0}\,{\Omega }_{f,k}(t){e}^{-ik{\omega }_{d}t}$ and $g(t)={\sum }_{k\ne 0}\,{\Omega }_{g,k}(t){e}^{-ik{\omega }_{d}t}\mathrm{}.$ The effective Hamiltonian acting on the system to leading order in large driving frequency is obtained by computing the stroboscopic Floquet-Magnus expansion generated by ${H}_{{\rm{fmod}}}$^26,48^. Assuming to use only terms oscillating at the fundamental harmonic ${\omega }_{d}$ in the control functions, the effective Hamiltonian to leading order in ${\omega }_{d}^{-1}$ is

12 $${H}_{{\rm{e}}{\rm{f}}{\rm{f}}}^{(1)}=\frac{1}{4{\omega }_{d}}(\begin{array}{ccc}|{\Omega }_{f,-1}{|}^{2}-|{\Omega }_{f,1}{|}^{2} & 0 & {\Omega }_{f,-1}{\Omega }_{g,1}-{\Omega }_{f,1}{\Omega }_{g,-1}\\ 0 & |{\Omega }_{f,1}{|}^{2}-|{\Omega }_{f,-1}{|}^{2}-(|{\Omega }_{g,1}{|}^{2}-|{\Omega }_{g,-1}{|}^{2}) & 0\\ {\Omega }_{f,-1}^{\ast }{\Omega }_{g,1}^{\ast }-{\Omega }_{f,1}^{\ast }{\Omega }_{g,-1}^{\ast } & 0 & |{\Omega }_{g,1}{|}^{2}-|{\Omega }_{g,-1}{|}^{2}\end{array}).$$

The diagonal elements in Eq. (12) represent the highest-order ac-Stark shifts induced by the drivings. For the effective Hamiltonian of Eq. (12) to match the counterdiabatic correction ${H}_{{\rm{cd}}}$, these diagonal entries must be zero while for the off-diagonal elements it must hold

13 $$\frac{1}{4{\omega }_{d}}[{\Omega }_{f,-1}{\Omega }_{g\mathrm{,1}}-{\Omega }_{f\mathrm{,1}}{\Omega }_{g,-1}]=i\frac{{\Omega }_{{\rm{cd}}}}{2}$$

and the complex-conjugated equation. Taking into account that ${\Omega }_{{\rm{cd}}}(t)$ is real-valued and non-negative with our choice of the phases of the STIRAP pulses, these constraints can be satisfied by taking ${\Omega }_{f\mathrm{,1}}={\Omega }_{f,-1}$, ${\Omega }_{g\mathrm{,1}}=-{\Omega }_{g,-1}$. Then, choosing also ${\Omega }_{f\mathrm{,1}}=i{\Omega }_{g,-1}={\Omega }_{d}$, the constraint (13) gives ${\Omega }_{d}(t)=\sqrt{{\omega }_{d}{\Omega }_{{\rm{cd}}}(t)}$ and one obtains the shortcut Hamiltonian of Eq. (6).

### fmod-STIRAP implementation in an optical excitation

We consider a possible experimental realization where an input laser intensity ${I}_{{\rm{in}}}$, corresponding to a Rabi frequency ${\Omega }_{{\rm{in}}}$ assumed up to 40 in units of $T$, is available for each branch of the three-level scheme. Each input laser is split into two beams each with intensity ${I}_{{\rm{in}}}\mathrm{/2}$ and Rabi frequency ${\Omega }_{{\rm{in}}}/\sqrt{2}$. These beams are processed through acousto-optical modulators in order to generate sidebands at frequencies ${\omega }_{d}$ and $-{\omega }_{d}$, with phases ${\phi }_{G}$ and ${\phi }_{G}+\pi \mathrm{/2}$, each modulator having an efficiency ${\alpha }_{m}$ up to 0.9. Recombining the outputs at the two different phases it is possible to realize an excitation with a carrier having Rabi frequency up to ${\Omega }_{0}T=40\sqrt{1-{\alpha }_{m}}$ and sidebands up to ${\Omega }_{d}^{{\rm{peak}}}T=20\sqrt{{\alpha }_{m}\mathrm{/(1}-{\alpha }_{m})}$.

### Relaxation

Energy relaxation is taken into account in the numerical simulations by using the following Lindblad master equation for the density matrix $\rho (t)$ of the system,

$$\frac{\partial \rho (t)}{\partial t}=-\,i\,[{H}_{{\rm{st}}}(t)+{H}_{{\rm{fmod}}}(t),\rho (t)]+\mathop{\sum }\limits_{k\mathrm{=0}}^{1}{\Gamma }_{k,k+1}({C}_{k}\rho (t){C}_{k}^{\dagger }-\frac{1}{2}\{{C}_{k}^{\dagger }{C}_{k},\rho (t)\})$$

with ${H}_{{\rm{st}}}(t)$ the STIRAP Hamiltonian (1), ${H}_{{\rm{fmod}}}$ the shortcut Hamiltonian (6), and collapse operators ${C}_{k}=|k\rangle \langle k+1|$. Our analysis considers both an optical and a microwave implementation. The first one involves a Rydberg STIRAP excitation, applied for instance in recent experiments^50^, using the parameters^51^ ${\Gamma }_{01}T\mathrm{=7}$ and ${\Gamma }_{12}T=3\cdot {10}^{-3}$ with a wide difference between the two relaxation rates. The second one is based on recent STIRAP implementations with superconducting circuits^15^, where ${\Gamma }_{01}T=0.017$, ${\Gamma }_{12}T=0.023$. For treating the case of systems with fast decoherence, we have also introduced artificial dephasing through the collapse operators

$${\overline{C}}_{k}=\sqrt{{\gamma }_{k}}|k\rangle \langle k|$$

where the rates ${\gamma }_{k}$ are taken in the range $0-100\times {\Gamma }_{12}$.

### Transfer times

#### Constant pulse

For a constant ${\sigma }_{y}$ pulse connecting states $|0\rangle$ and $|2\rangle$ of the three-level system with Rabi frequency ${\Omega }_{\pi }$, described by the Hamiltonian ${H}_{\pi }=i{\Omega }_{\pi }\mathrm{/2[}|2\rangle \langle 0|\,-\,|0\rangle \langle 2|],$ the transfer time between two states $|{\psi }_{i}\rangle$ and $|{\psi }_{f}\rangle$, separated by an angle ${\Theta }_{f,i}$ within the $|0\rangle$-$|2\rangle$ subspace, is^36^

14 $${\tau }_{\pi }=\frac{2{\Theta }_{f,i}}{{\Omega }_{\pi }}=\frac{2arccos|\langle {\psi }_{f}|{\psi }_{i}\rangle |}{{\Omega }_{\pi }}.$$

Parametrizing the states like ${\psi }_{k}=\,\cos \,{\theta }_{k}|0\rangle +{e}^{i{\phi }_{k}}\,\sin \,{\theta }_{k}|2\rangle$, one can write the initial occupation probability of state $|0\rangle$ as ${p}_{0}^{(i)}={cos}^{2}{\theta }_{i}$ and the final occupation probability of state $|2\rangle$ as ${p}_{2}^{(f)}={sin}^{2}{\theta }_{f}$. In this way, ${\Theta }_{f,i}={\theta }_{f}-{\theta }_{i}$ and Eq. (14) can be restated in terms of the probabilities ${p}_{0}^{(i)}$ and ${p}_{2}^{(f)}$ as ${\tau }_{\pi }=2[arcsin\sqrt{{p}_{2}^{(f)}}-arccos\sqrt{{p}_{0}^{(i)}}]/{\Omega }_{\pi }\mathrm{}.$ The pulse area is simply ${\int }_{{t}_{i}}^{{t}_{f}}\,{\Omega }_{\pi }dt={\Omega }_{\pi }{\tau }_{\pi }$.

#### cd-STIRAP

Since, under cd-STIRAP, the system instantaneously follows the dark state with unit fidelity, the occupation probabilities of states $|0\rangle$ and $|2\rangle$ can be expressed in terms of the mixing angle, ${p}_{0}(t)={\cos }^{2}\,\theta (t)$, ${p}_{2}(t)={\sin }^{2}\,\theta (t)$. For the Gaussian pulses of Eq. (2), these expressions can be inverted for finding the time as a function of the populations, by using in the intermediate steps the explicit expressions of the pulses and of the mixing angle. The result is

$$t({p}_{0}=p)=\frac{1}{{\Omega }_{{\rm{c}}{\rm{d}}}^{{\rm{p}}{\rm{e}}{\rm{a}}{\rm{k}}}}\,\log \,[\sqrt{\frac{1-p}{p}}];\,t({p}_{2}=p)=\frac{1}{{\Omega }_{{\rm{c}}{\rm{d}}}^{{\rm{p}}{\rm{e}}{\rm{a}}{\rm{k}}}}\,\log \,[\sqrt{\frac{p}{1-p}}].$$

The difference ${\tau }_{{\rm{cd}}}=t({p}_{2}={p}_{2}^{(f)})-t({p}_{0}={p}_{0}^{(i)})$ then gives the total transfer time, which is thus completely governed by the peak value of the cd Rabi frequency. Since the dark state matches exactly the states $|0\rangle$ and $|2\rangle$ only asymptotically, the transfer time is consistently divergent for unit initial and final occupations. The pulse area for a given transfer is the same as that for a constant-Rabi pulse^19,28^.

#### fmod-STIRAP

For determining the transfer time of the fmod-STIRAP protocol, the dynamics of the populations ${p}_{0}$ and ${p}_{2}$ of the bare states, exemplified in Fig. 2, is first of all fitted using a sigmoid function of the form $\exp \,(\mu t+\nu \mathrm{)/[1}+\exp [(\mu t+\nu \mathrm{)]}.$ Once the parameters $\mu$ and $\nu$ are estimated, one can determine the time at which the population ${p}_{n}$ of state $|n\rangle$– $n=0,\,2$ – is equal to ${p}_{n}=p$, which is

$$t({p}_{n}=p)=\frac{1}{{\mu }_{n}}[\log (\frac{p}{1-p})-{\nu }_{n}].$$

Finally, the transfer time is computed as ${\tau }_{{\rm{fmod}}}=t({p}_{2}={p}_{2}^{(f)})-t({p}_{0}={p}_{0}^{(i)})$ for the initial probability ${p}_{0}^{(i)}$ of being in state $|0\rangle$ and final probability ${p}_{2}^{(f)}$ of being in state $|2\rangle$. The pulse area for each of the required sidebands is

15 $${\int }_{{t}_{i}}^{{t}_{f}}|{\Omega }_{d}(t)|dt=2i\sqrt{\frac{{\omega }_{d}}{{\Omega }_{{\rm{c}}{\rm{d}}}^{{\rm{p}}{\rm{e}}{\rm{a}}{\rm{k}}}}}{F[\frac{i{\Omega }_{{\rm{c}}{\rm{d}}}^{{\rm{p}}{\rm{e}}{\rm{a}}{\rm{k}}}t}{2},\,2]|}_{{t}_{f}}^{{t}_{i}},$$

where the last equality holds for the Gaussian pulses of Eq. (2) and $F[\phi ,m]$ is the elliptic integral of the first kind^52^. In producing Fig. 4b, the result of Eq. (15) is multiplied by four for taking into account all the sidebands.
